# Expanding the Arsenal of FGFR Inhibitors: A Novel Chloroacetamide Derivative as a New Irreversible Agent With Anti-proliferative Activity Against FGFR1-Amplified Lung Cancer Cell Lines

**DOI:** 10.3389/fonc.2019.00179

**Published:** 2019-03-26

**Authors:** Claudia Fumarola, Nicole Bozza, Riccardo Castelli, Francesca Ferlenghi, Giuseppe Marseglia, Alessio Lodola, Mara Bonelli, Silvia La Monica, Daniele Cretella, Roberta Alfieri, Roberta Minari, Maricla Galetti, Marcello Tiseo, Andrea Ardizzoni, Marco Mor, Pier Giorgio Petronini

**Affiliations:** ^1^Department of Medicine and Surgery, University of Parma, Parma, Italy; ^2^Department of Food and Drug, University of Parma, Parma, Italy; ^3^Medical Oncology Unit, University Hospital of Parma, Parma, Italy; ^4^Italian Workers' Compensation Authority (INAIL) Research Center, Parma, Italy; ^5^Department of Medicine and Surgery, Center of Excellence for Toxicological Research, University of Parma, Parma, Italy; ^6^Division of Medical Oncology, Sant'Orsola-Malpighi University Hospital and Alma Mater University of Bologna, Bologna, Italy

**Keywords:** FGFR, irreversible inhibitors, drug design, lung cancer, SQCLC, cancer drug resistance

## Abstract

Fibroblast Growth Factor Receptors (FGFR1–4) have a critical role in the progression of several human cancers, including Squamous Non-Small-Cell Lung Cancer (SQCLC). Both non-selective and selective reversible FGFR inhibitors are under clinical investigation for the treatment of patients with tumors harboring FGFR alterations. Despite their potential efficacy, the clinical development of these drugs has encountered several challenges, including toxicity, and the appearance of drug resistance. Recent efforts have been directed at development of irreversible FGFR inhibitors, which have the potential to exert superior anti-proliferative activity in tumors carrying FGFR alterations. With this in mind, we synthetized, and investigated a set of novel inhibitors possessing a warhead potentially able to covalently bind a cysteine in the P-loop of FGFR. Among them, the chloroacetamide UPR1376 resulted able to irreversible inhibit FGFR1 phosphorylation in FGFR1 over-expressing cells generated from SQCLC SKMES-1 cells. In addition, this compound inhibited cell proliferation in FGFR1-amplified H1581 cells with a potency higher than the reversible inhibitor BGJ398 (infigratinib), while sparing FGFR1 low-expressing cells. The anti-proliferative effects of UPR1376 were demonstrated in both 2D and 3D systems and were associated with the inhibition of MAPK and AKT/mTOR signaling pathways. UPR1376 inhibited cell proliferation also in two BGJ398-resistant cell clones generated from H1581 by chronic exposure to BGJ398, although at concentrations higher than those effective in the parental cells, likely due to the persistent activation of the MAPK pathway associated to *NRAS* amplification. Combined blockade of FGFR1 and MAPK signaling, by UPR1376 and trametinib respectively, significantly enhanced the efficacy of UPR1376, providing a means of circumventing resistance to FGFR1 inhibition. Our findings suggest that the insertion of a chloroacetamide warhead on a suitable scaffold, as exemplified by UPR1376, is a valuable strategy to develop a novel generation of FGFR inhibitors for the treatment of SQCLC patients with FGFR alterations.

## Introduction

The Fibroblast Growth Factor Receptor (FGFR) tyrosine kinase (TK) family consists of four members (FGFR1-4), activated through 22 different FGF ligands, which regulate multiple biological processes, including cell proliferation, migration, differentiation, apoptosis, metabolism, and angiogenesis (1). Upon ligand binding, FGFRs dimerize and activate a complex downstream signaling, including mitogen activated protein kinase (MAPK), phosphoinositide-3-kinase (PI3K)/Akt, and signal transducer and activator of transcription (STAT) pathways (2). Deregulation of FGFR signaling, via gene amplification, overexpression, point mutations or chromosomal translocations has been implicated in several human cancers, such as lung, breast, prostate, and endometrial cancers (3). In lung cancer, FGFR1 amplification is observed in 9–20% of cases of the Squamous Non-Small-Cell Lung Cancer (SQCLC) histotype (3–5). SQCLC is a challenging disease characterized by a marked mutational complexity that renders difficult the development of effective molecular-targeted therapies. Although immunotherapy has recently shown great promise (6–9), platinum-based regimens are still the standard of care 1st-line therapy for the majority of patients. Therefore, the potential of FGFR signaling as a therapeutic target in SQCLC warrants continued exploration to provide a valuable treatment option at least for the subset of patients carrying FGFR alterations.

**Figure 1:**
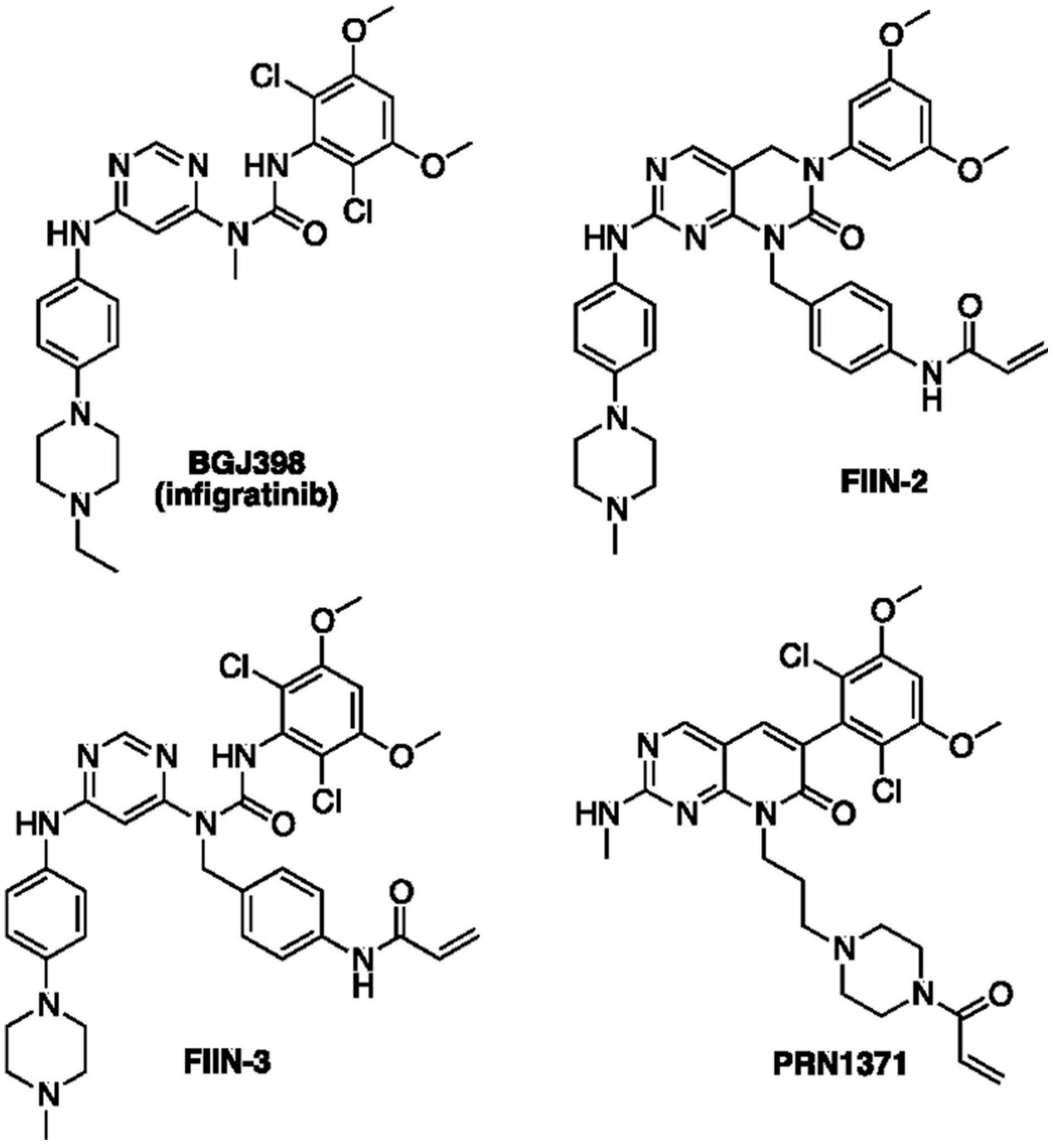
Chemical structures of relevant inhibitors of FGFR.

Since the identification of FGFR as a relevant target for cancer therapy, a number of FGFR inhibitors have been developed and some are currently under clinical evaluation in various FGFR-related tumors (10). The most clinically advanced compounds are non-selective FGFR TKIs, such as dovitinib and the Food and Drug Administration (FDA)-approved ponatinib, which target other related TKs, including Vascular Endothelial Growth Factor Receptors (VEGFRs) and Platelet Derived Growth Factor Receptors (PDGFRs). Despite their potential efficacy, the clinical success of these drugs is limited by the increased adverse side effects associated with their broad inhibitory activity (2). More recently, potent and highly selective FGFR inhibitors, such as BGJ398 (infigratinib, Figure 1) and others, i.e., AZD-4547 (11) and JNJ-42756493 [erdafitinib (12)], have shown promising pre-clinical anti-tumor activity, thereby entering into clinical investigation. However, although these agents display a more favorable safety profile as compared to FGFR non-selective inhibitors (13, 14), their clinical efficacy has not been unequivocally demonstrated, with the less encouraging results obtained in patients with FGFR1 amplification (15, 16). Moreover, as seen with other RTK inhibitors, the clinical benefit of FGFR-targeted therapies is inevitably hampered by the emergence of acquired resistance (17), arising from activation of by-pass signaling mechanisms or selection of gatekeeper mutations that abrogate the drug inhibitory activity on the receptor.

Inhibitors able to engage FGFR with an irreversible mechanism of action have the potential to overcome the effect of genetic alterations emerging in FGFR inhibitor-treated tumors (18). The clinical experience gained in the field of EGFR and BTK kinases (19) have shown that compounds of this kind are more therapeutically effective than their reversible analogs, in light of the following properties: (i) irreversible inhibitors do not readily dissociate from the engaged target thanks to the formation of a covalent bond; (ii) they cannot be displaced by ATP once the covalent bond with the target is formed; (iii) they sustain prolonged inhibition of the signaling pathways also after elimination from the cells, as the inhibitory process can be reverted only through the *de novo* synthesis of the protein (20, 21). Recent attempts to develop irreversible inhibitors of FGFR have led to the identification of acrylamide-based compounds such as FIIN-2/FIIN-3 (18) and PRN1371 (22) (Figure 1), which alkylate a non-catalytic cysteine present in the P-loop of FGFR isoforms (Cys488 in FGFR1). These compounds show excellent anti-proliferative activity in a variety of lung cancer cell lines with a potency comparable or superior to that of the clinical candidate BGJ398 (18, 22). These compounds also inhibited the growth of SQCLC cell lines resistant to BGJ398, emerging as potentially useful for treating FGFR-dependent cancers, such as cholangiocarcinoma or metastatic urothelial cancer, after progression (23). In the present work, we report and characterize a focused set of FGFR inhibitors based on the 1-(4-aminobenzyl)-pyrimido[4,5-*d*]pyrimidin-2-one core of FIIN-2, equipped with warheads different from acrylamide, with the aim to expand the arsenal of available irreversible agents targeting FGFR.

## Materials and Methods

### Cell Culture

The human NSCLC cell lines SKMES-1 and H1581 were purchased from the American Type Culture Collection (ATCC, Manassas, VA); ATCC authenticates the phenotypes of these cell lines on a regular basis. FGFR1-over-expressing LENTI-4 cells were generated from FGFR1-low-expressing SKMES-1 cells using a lentiviral vector system, as previously described (24). BGJ398-resistant cells were generated from H1581 cells by continuous culturing with increasing concentrations of BGJ398 up to 1 μM, and were routinely maintained in the presence of 1 μM BGJ398. All the cells were cultured in RPMI 1640 medium, supplemented with 2 mM L-glutamine, 10% fetal calf serum (FCS), and 1% penicillin/streptomycin solution, and maintained at 37°C in a humidified atmosphere of 5% CO_2_ and 95% air.

### Compounds

BGJ398 was provided by Novartis International AG (Basel, Switzerland). FIIN-2 was purchased from Selleck Chemicals (Houston, TX). UPR1371-76 were prepared as described in the Supplementary Material. All the drugs were dissolved in DMSO (Sigma-Aldrich, Saint Louis, MO) and diluted in fresh medium before use. The final concentration of DMSO in medium never exceeded 0.1 % (v/v) and equal amounts of the solvent were added to control cells.

### Western Blotting Analysis

Procedures for protein extraction, solubilization, and protein analysis by 1-D PAGE are described elsewhere (25). Antibodies against p-FGFR^Tyr653/654^, FGFR1, p-ERK1/2^Thr202/Tyr204^, ERK1/2, p-mTOR^Ser2448^, mTOR, p-AKT^ser473^, AKT, p-P70S6K^Thr389^, P70S6K were from Cell Signaling Technology (Beverly, MA); the antibody against actin was from Sigma-Aldrich. HRP-conjugated secondary antibodies were from Pierce (Rockford, IL) and chemiluminescence system (ImmobilionTM Western Chemiluminescent HRP Substrate) was from Millipore (Temecula, CA). The chemiluminescent signal was acquired by C-DiGit® Blot Scanner (LI-COR Biotechnology, Lincoln, NE). Where indicated, phosphorylation levels were quantified by Image Studio™ Software (LI-COR Biotechnology), and normalized to the corresponding protein levels.

### Autophosphorylation and Washout Assay

The cells, serum-starved for 24 h, were pre-incubated for 1 h with the compounds at 1 μM concentration and then stimulated for 15 min with 25 ng/ml FGF2 (Miltenyi Biotec, Bergisch Gladbach, Germany). For the washout assay, the cells, pre-incubated with the compounds for 1 h, were extensively washed with PBS, and then maintained in drug-free medium for additional 8 h before stimulation with FGF2 for 15 min. The cells were lysed and equal amounts of cell protein extracts were analyzed by Western blotting using a phospho-FGFR antibody. Membranes were stripped and reprobed with anti-FGFR1 antibody.

### Determination of Intracellular Concentrations of Selected Compounds

The cells were plated at 1 × 10^6^ cells cells/dish (25 cm^2^) density. After 24 h, BGJ398, FIIN-2 or UPR1376 were added to the culture medium (titled concentration: 1 μM with DMSO 0.1% v/v). At the end of incubation, the compounds were removed from the extracellular medium by washing the cells for three times with 1 mL aliquot of fresh medium. The cells were treated using absolute EtOH (1.1 mL at 4°C) to obtain intracellular extracts. The final cell extracts were centrifuged (4°C, 10,000 g, 5 min) and collected. A fixed volume of ethanolic extract was evaporated to dryness, dissolved in LC eluent and injected into the LC/MS system for quantitative measurement (see Supplementary Material). Cell proteins were quantified after solubilization in NaOH 0.5 N (2 mL/25 cm^2^ dish) by the Bradford method.

### Analysis of Cell Proliferation

Cell proliferation was evaluated by tetrazolium dye [3-(4,5-dimethylthiazol-2-yl)-2,5-diphenyltetrazolium bromide (MTT), Sigma Aldrich] assay, as previously described (26). The concentration that inhibits 50% of cell proliferation (IC_50_) was extrapolated from the dose-response curves calculated from experimental points using Graph-Pad Prism version 6.0 software (GraphPad Software, San Diego, CA). The nature of the interaction between UPR1376 and trametinib was calculated using the Bliss additivism model (27). A theoretical dose-response curve was calculated for combined inhibition using the equation of Bliss = EA + EB-EA^*^EB, where EA and EB are the percent of inhibition vs. control obtained by UPR1376 (A) and trametinib (B) alone and the E Bliss is the percent of inhibition that would be expected if the combination was exactly additive. If the combination effect is higher than the expected Bliss equation value, the interaction is synergistic, while if the effect is lower, the interaction is antagonistic. Otherwise, the effect is additive and there is no interaction between the drugs.

### Spheroid Generation

Spheroids were generated using LIPIDURE®-COAT PLATE A-U96 (NOF Corporation, Tokyo, Japan) as previously described (28). Briefly, 500 cells were seeded in RPMI 1,640 medium and after 3 days (T0) the spheroids were treated with BGJ398, FIIN-2 or UPR1376 for further 10 days. The effect of the drugs was evaluated in term of volume changes using the Nikon Eclipse E400 Microscope with digital Net camera. The volume of spheroids was measured [D = (Dmax+Dmin)/2; V = 4/3π(D/2)3] using SpheroidSizer, a MATLAB-based and open-source software application (29).

### Analysis for the Presence of FGFR1 p.V561M Mutation

Genomic DNA was extracted from the cells using the QIAamp DNA Mini Kit (Qiagen Inc., Valencia, CA, USA), and stored at −20°C until use. Primers were designed in FGFR1 exon 13 with Primer3 software.

FGFR1-exon13 Forward 5′ tgctcgggaattttctggac 3′

FGFR1-exon13 Reverse 5′ caacgccaccacaagatgat 3′

Exon 13 of FGFR1 gene (GeneBank accession number NM_023110) was amplified for each sample by Polymerase Chain Reaction (PCR) using AmpliTaq Gold DNA Polymerase (Applied Biosystems) following manufacturer's protocol. PCR conditions were the following: 95°C for 10 min, 15 cycles with touch down protocol with annealing temperature (TA) from 63 to 56°C and 35 cycles with TA at 56°C. A final step of 10 min at 72°C was performed. Genomic DNA was sequenced using a CEQ Dye-Terminator Cycle Sequencing kit (Beckman Coulter Inc., Miami, FL, USA) according to the manufacturer's protocol. Sequence alignments were performed with the DNAStar program (DNAStar Inc., Madison, WI, USA). All the sequence reactions were performed using a CEQ XL2000 DNA Analysis System (Beckman Coulter).

### *NRAS* Amplification

The analysis of *NRAS* amplification was performed by a digital droplet PCR (ddPCR), using a Copy Number Assay (BioRad®, Hercules, CA) following the manufacturer's instructions.

NRAS assay (dHsaCP1000493, BioRad) was labeled in FAM, and reference assay AP3B1 (dHsaCP2500348), chosen among recommended reference assays by BioRad, was labeled in VIC.

### Statistical Analysis

Statistical analyses were carried out using Graph-Pad Prism version 6.0 software. Statistical significance of differences among data was estimated by Student's *t*-test and *p*-values are indicated where appropriate in the figures and in their legends *p* < 0.05 were considered significant.

## Results

### Chemistry

**Figure 2:**
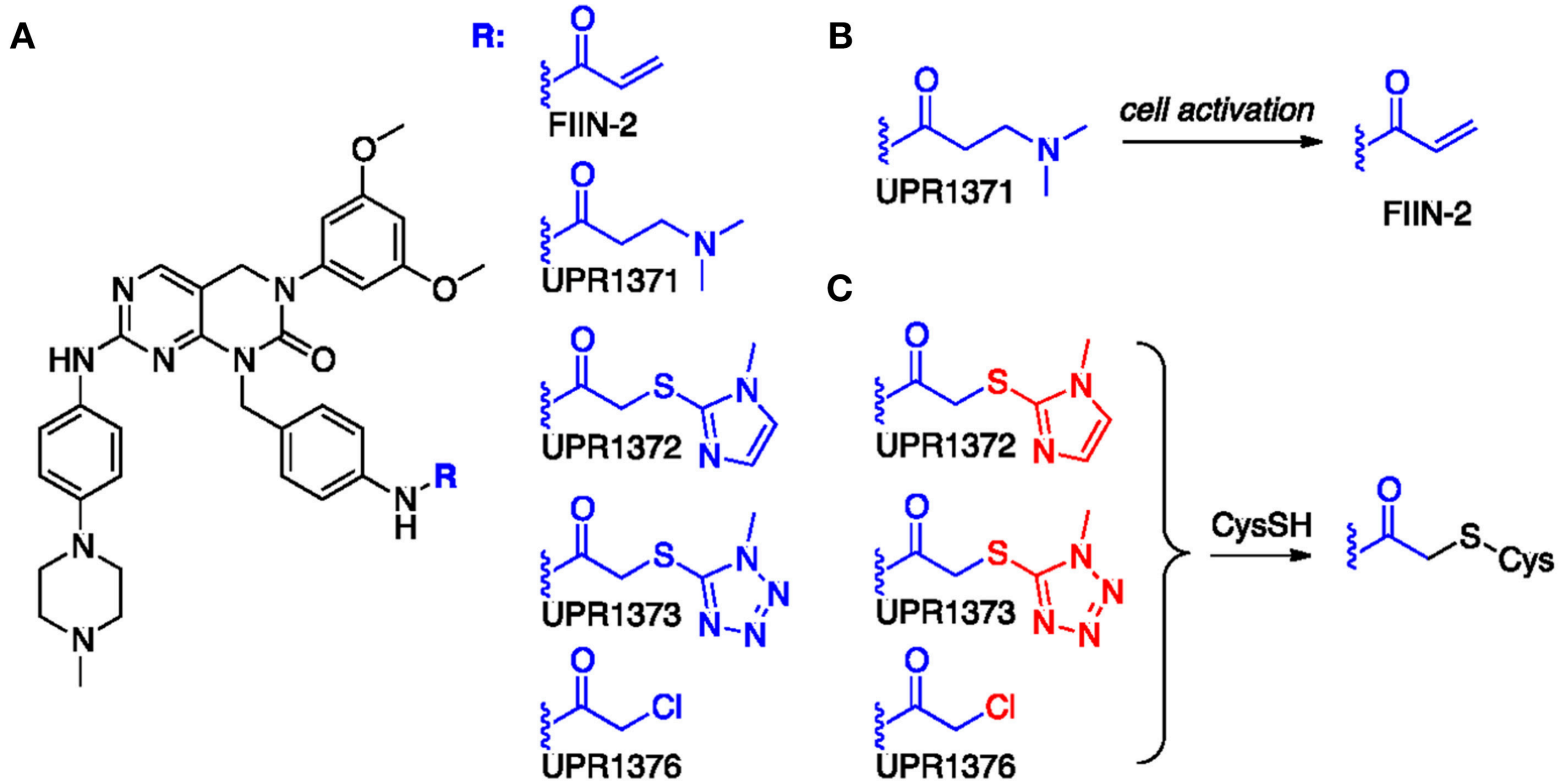
**(A)** Chemical formulas of tested compounds. **(B)** Hypothesized conversion of 3-APA group of UPR1371 in acrylamide of FIIN-2. **(C)** Putative mechanism of action for acetamide derivatives UPR1372, UPR1373 and UPR1376. The leaving group installed on the acetamide fragment is colored in red.

Starting from the structure of FIIN-2 (Figure 2A), we synthesized a small set of new potential FGFR inhibitors replacing the terminal acrylamide installed on the aminobenzyl pendant of this compound with other chemical groups. Our design strategy was based on two distinct approaches. With the first, we masked the acrylamide warhead by preparing the 3-aminopropanamide (3-APA) derivative UPR1371. The 3-APA group is not itself capable to covalently bind nucleophiles, but it can undergo selective activation in the intracellular environment of cancer cells (30), releasing the acrylamide group (Figure 2B). With the second, the acrylamide was replaced by activated acetamides, i.e., by electrophilic groups potentially able to alkylate the P-loop cysteine of FGFR isoforms by nucleophilic substitution (Figure 2C), differently from acrylamides which still alkylates cysteine residues, but with a different mechanism, namely a Michael addition. This is the case of 2-((1*H*-imidazol-2-yl)thio)acetamide UPR1372, 2-((1*H*-tetrazol-5-yl)thio)acetamide UPR1373 and 2-chloroacetamide UPR1376. Acetamides of this kind have been recently used by our group to obtain irreversible inhibitors of EGFR (31–33) that also possesses a critical cysteine at the ATP binding site. The procedures employed to synthesize the title compounds, along with their chemical characterization, are reported in the Supplementary Material.

### UPR1376 Inhibits FGFR1 Auto-Phosphorylation Irreversibly in FGFR1 Over-Expressing SQCLC Cells

**Figure 3:**
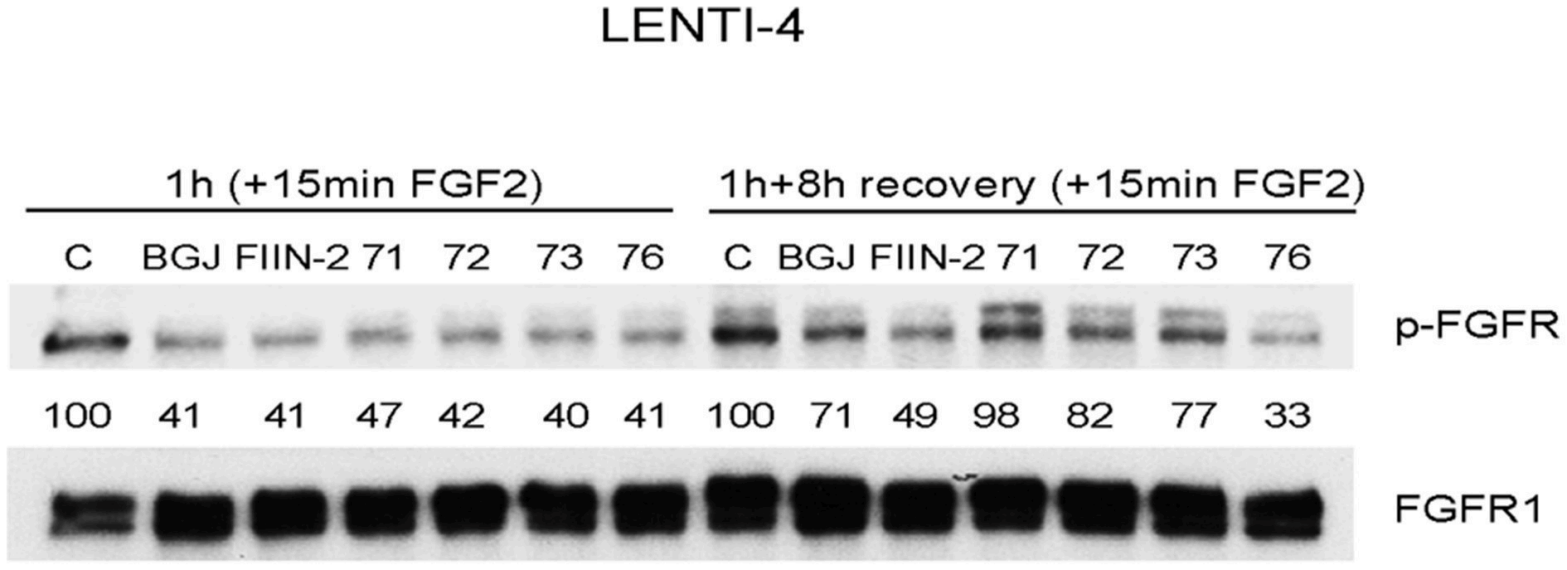
Inhibitory effects of UPR1371-UPR1376 on FGFR1 auto-phosphorylation in LENTI-4 cells. LENTI-4 cells, serum-deprived for 24 h, were pre-treated with 1 μM BGJ398, FIIN-2 or the newly synthesized FGFR inhibitors (UPR1371-UPR1376). After 1 h, the cells were stimulated with FGF2 for further 15 min, or extensively washed with PBS and stimulated with FGF2 for 15 min after 8 h of incubation in fresh drug-free medium. At the end of the treatments, the cells were lysed and the protein extracts were analyzed by Western blotting for FGFR1 auto-phosphorylation. Results are representative of three independent experiments. The immunoreactive spots were quantified by densitometric analysis, ratios of p-FGFR/total FGFR were calculated and values, expressed as percent vs. control, are reported.

The newly synthesized compounds were analyzed for their ability to inhibit FGFR auto-phosphorylation in LENTI-4 cells, a FGFR1-over-expressing cell model generated in our lab from SQCLC FGFR1 low-expressing SKMES-1 cells. As indicated in Figure 3, all the compounds down-regulated FGF2-induced phosphorylation of FGFR1 after 1 h of treatment, with an efficacy comparable to that shown by the reversible FGFR inhibitor BGJ398 and the irreversible reference inhibitor FIIN-2. To test the irreversible activity of UPR1371-UPR1376 compounds, wash-out experiments were performed in which LENTI-4 cells were exposed to the drugs for 1 h, extensively washed with PBS, and then incubated in drug-free medium for further 8 h before stimulation with FGF2. While UPR1371, 1372, and 1373 failed to maintain FGFR1 inhibition, allowing an almost complete recovery of auto-phosphorylation after 8 h, UPR1376 was even more effective than FIIN-2 in sustaining the inhibition of FGFR1 auto-phosphorylation (Figure 3), suggesting its ability to covalently interact with the receptor. As expected, FGFR1 auto-phosphorylation was reversibly inhibited by BGJ398 and recovered 8 h after BGJ398 withdrawal, although the restoration was not complete, presumably because of the efficient trapping of the drug into the cells (34).

To further characterize the biological activity of the most interesting compounds (BGJ398, FIIN-2, and UPR1376), we measured their intracellular level in LENTI-4 cells immediately after 1 h of exposure to each inhibitor (nominal concentration of 1 μM) or 8 h after washing the inhibitor from the extracellular medium by LC/MS (see Supplementary Material). Measured concentrations are summarized in Table 1. At both time points, the chloroacetamide derivative UPR1376 displayed an intracellular concentration significantly lower than BGJ398 or FIIN-2. This could be ascribed to its lower metabolic stability, as indicated by the residual compound concentration measured in the cellular medium after 8 h, i.e., 69.4 ± 2.5 % for UPR1376, 90.2 ± 9.0 for BGJ398, and 85.1 ± 2.8 % for FIIN-2. In spite of a lower intracellular concentration, UPR1376 resulted able to inhibit FGFR auto-phosphorylation as effectively as BGJ398 and FIIN-2 at 1 h and even more than these two reference compounds 8 h after compound removal. This suggests that UPR1376 is more potent than BGJ398 and FIIN-2 in the auto-phosphorylation assay.

**Table 1:** Intracellular levels of selected FGFR inhibitors measured in LENTI-4 cells by LC/MS.

**Cpds**	**Intracellular concentration (pmol/mg prot)**	
	**1 h**	**8h**
BGJ398	1,843	52
FIIN-2	1,132	95
UPR1376	109	4

*LENTI-4 cells were incubated with 1 μM solution of titled compound for 1 h. Intracellular content was measured immediately after incubation and 8 h after removal of the compound from the medium by extensive washing by LC/MS. Concentrations are expressed as pmol of compound per mg of protein, determined using Bradford assay. Results are representative of two independent experiments*.

### UPR1376 Down-Regulates FGFR1 Signaling and Inhibits Cell Proliferation in FGFR1-Amplified H1581 Cells

**Figure 4:**
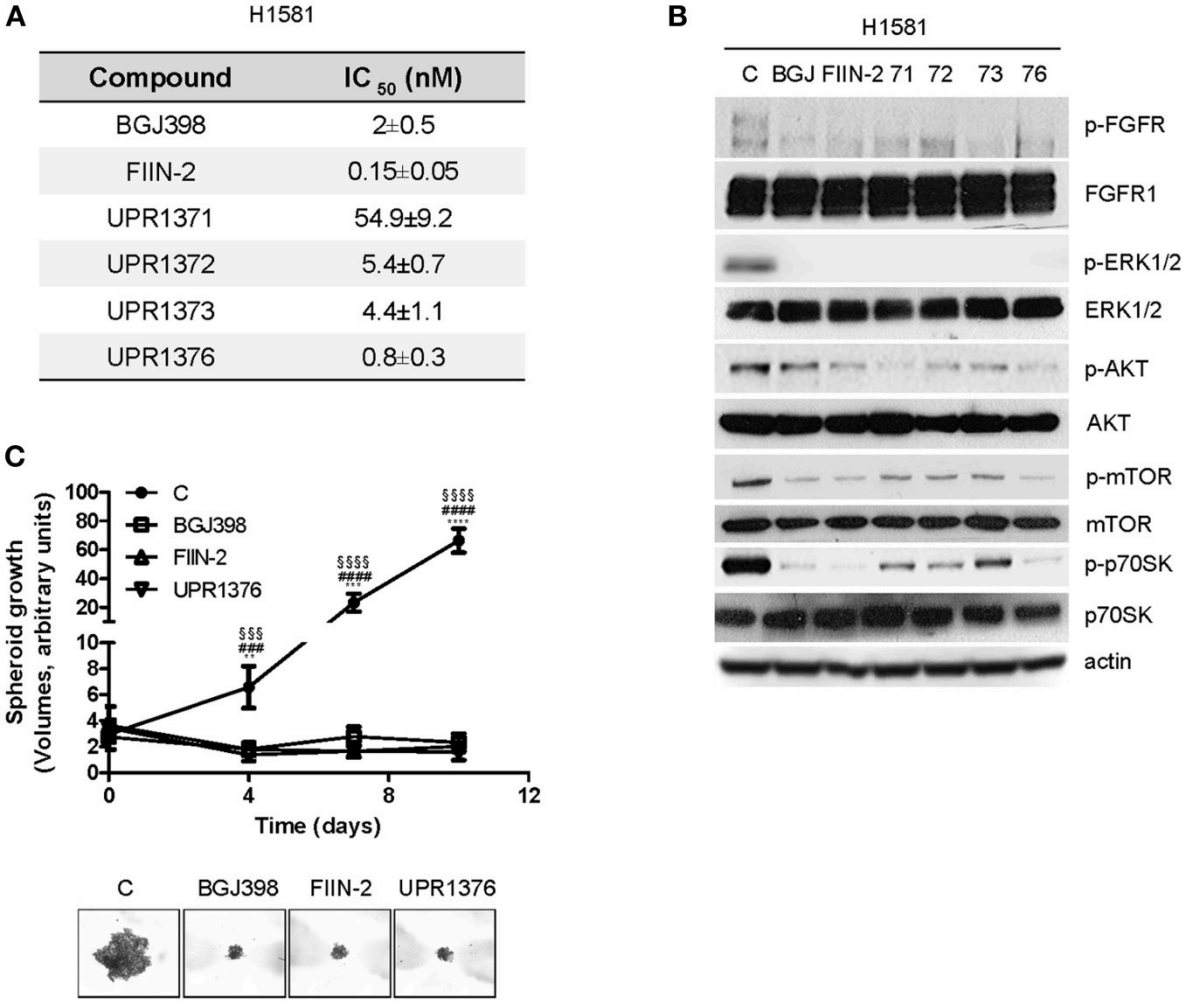
Effects of BGJ398, FIIN-2, and UPR1371-UPR1376 on cell growth and FGFR1 signaling in H1581 cells. **(A)** H1581 cells were treated with increasing concentrations of the FGFR inhibitors (0.001 nM-10 μM) and after 72 h cell proliferation was assessed by MTT assay. The IC_50_ values shown are means ± SD of at least three independent experiments. **(B)** H1581 cells were treated with the FGFR inhibitors at 1 μM for 6 h, and then protein lysates were analyzed by Western blotting for the indicated proteins. Results are representative of two independent experiments. **(C)** H1581 cells were grown as tumor spheroids in the absence or presence of BGJ398, FIIN-2, or UPR1376 at 10 nM. Spheroid volumes were measured at 3 days after seeding (T0), and after 4, 7, or 10 days of culture. The data are means ± SD of four independent determinations. **p < 0.01, *** *p* < 0.001, **** *p* < 0.0001 for BGJ398 vs. control; ^###^ *p* < 0.001, ^####^ *p* < 0.0001 for FIIN-2 vs. control; ^§§§^ *p* < 0.001, ^§§§§^ *p* < 0.0001 for UPR1376 vs. control. Representative images of tumor spheroids at 10 days are shown.

The anti-tumor activity of UPR1371-UPR1376 was then evaluated in the NSCLC large cell carcinoma H1581 cell line, a cell model that harbors focal amplification of FGFR1 and is exquisitely sensitive to FGFR1 inhibition. All the compounds significantly inhibited cell proliferation with IC_50_ values in the nM range (Figure 4A). However, UPR1371 was the least effective, showing an IC_50_ value of ~55 nM; UPR1376 again demonstrated high efficacy, inhibiting cell proliferation with an IC_50_ value lower than that obtained with BGJ398 treatment. These growth-inhibitory effects were associated with the inhibition of FGFR1 phosphorylation, with consequent down-regulation of downstream signaling (Figure 4B). In particular, all the compounds were as effective as BGJ398 and FIIN-2 in inhibiting the MAPK pathway, as indicated by the complete dephosphorylation of ERK1/2 proteins, whereas the AKT pathway, with its downstream components mTOR and p70S6K, was more strongly down-regulated by UPR1376. Since UPR1376 appeared more effective than BGJ398 in two dimensional (2D) cultures, we evaluated its anti-proliferative activity also in three dimensional (3D) systems. As shown in Figure 4C, we demonstrated that not only BGJ398 and FIIN-2, but also UPR1376 completely inhibited the growth of tumor spheroids generated from H1581 cells, confirming its efficacy as an inhibitor of FGFR1-dependent cell growth.

### Generation and Characterization of BGJ398-Resistant H1581-Derived Cell Clones

**Figure 5:**
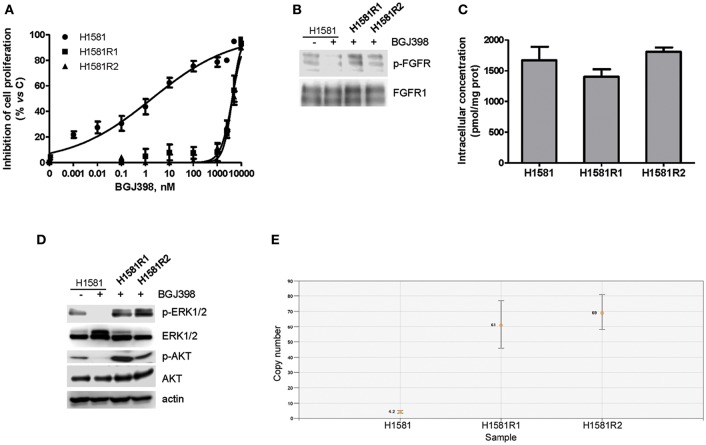
Characterization of BGJ398-resistant cell clones generated from H1581 cells. **(A)** H1581, H1581R1, and H1581R2 cells were incubated with increasing concentrations of BGJ398 (0.001 nM−10 μM). After 72 h, cell proliferation was assessed by MTT assay. The data are expressed as percent inhibition of cell proliferation vs. control. **(B)** H1581, H1581R1, and H1581R2 cells were incubated in the absence or presence of 1 μM BGJ398, and after 6 h protein lysates were analyzed by Western blotting for FGFR1 phosphorylation. **(C)** H1581, H1581R1, and H1581R2 cells were incubated with 1 μM BGJ398. Intracellular compound content was measured after 6 h by LC/MS. Concentrations are expressed as pmol of compound per mg of protein, determined using Bradford assay. **(D)** H1581 cells and H1581R clones were treated as in B and protein lysates were analyzed by Western blotting for the indicated proteins. **(E)** Genomic DNA was extracted from H1581 cells and H1581R clones and analyzed for the presence of *NRAS* amplification by ddPCR. Results in A and C are means ± SD of three independent experiments. Results in B and D are representative of three independent experiments.

The efficacy of the newly synthesized compounds was also evaluated in BGJ398-resistant cell clones generated from H1581 cells. Continuous exposure of H1581 cells to 50 nM BGJ398 initially led to the inhibition of cell proliferation associated with cell death. During culture, the concentration of BGJ398 was gradually increased up to 1 μM, and after 3 months of continuous treatment the selective pressure finally led to the emergence of cells no longer sensitive to the drug. Two independent cell clones were selected (H1581R1 and H1581R2), which, in contrast with the parental cell line, could grow in the presence of 1 μM BGJ398 and showed an IC_50_ value for cell proliferation > 4 μM (Figure 5A). As shown in Figure 5B, resistance of these cell clones to BGJ398 was associated with a persistent phosphorylation of FGFR1 despite the presence of BGJ398, in contrast with the almost complete inhibition induced by the drug in sensitive H1581 parental cells. We therefore performed Sanger sequencing of PCR products from H1581R cell clones to evaluate whether the resistance to FGFR1 inhibition was due to the presence of the V561M mutation at the gatekeeper residue located in the ATP-binding pocket of the receptor (35–37). However, neither of the two clones harbored such mutation (not shown), although the presence of other drug-resistant mutations at the level of the FGFR1 receptor cannot be ruled out. In addition, we excluded the activation of efflux pumps as a mechanism of resistance leading to a reduced accumulation of BGJ398 in H1581R clones; indeed, no difference in the intracellular concentration of the drug emerged between the resistant clones and the parental cells (Figure 5C).

Interestingly, both the AKT and MAPK pathways remained activated in H1581R clones in the presence of BGJ398 in contrast with the parental cells (Figure 5D); in addition, we found that *NRAS* was amplified in both clones, i.e., 61 copies for H1581R1 and 69 copies for H1581R2 cells vs. 4.2 copies for H1581 cells (Figure 5E), likely contributing to the resistant phenotype.

### UPR1376 Inhibits Cell Growth in H1581R Cell Clones and This Effect Is Enhanced by the Combination With the MEK1/2 Inhibitor Trametinib

**Figure 6:**
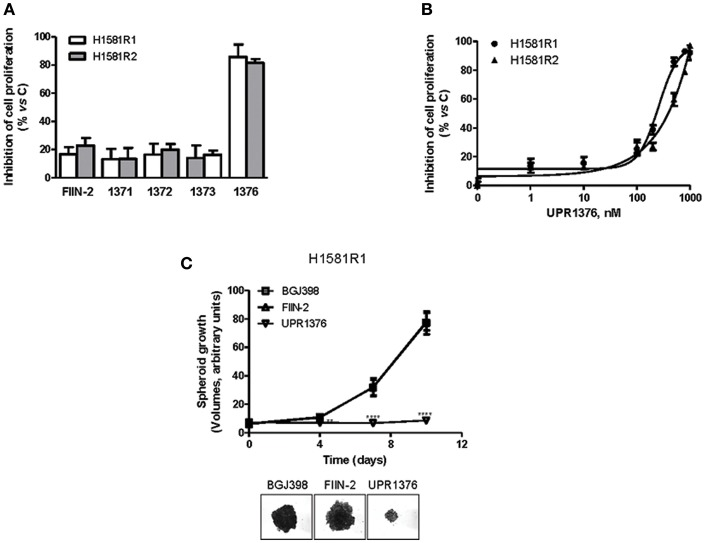
Effects of UPR1371-UPR1376 on cell growth in BGJ398-resistant H1581R1 and H1581R2 cell clones. **(A)** H1581R1 and H1581R2 cells were incubated with FIIN-2 reference inhibitor or UPR1371-UPR1376 compounds at 1 μM. After 72 h, cell proliferation was assessed by MTT assay. **(B)** H1581R1 and H1581R2 cells were incubated with increasing concentrations of UPR1376 (1–1,000 nM). After 72 h, cell proliferation was assessed by MTT assay. **(C)** H1581R1 cells were grown as tumor spheroids in the presence of 1 μM BGJ398, FIIN-2 or UPR1376. Spheroid volumes were measured at 3 days after seeding (T0), and after 4, 7, or 10 days of culture. **p < 0.01, ****p < 0.0001 vs. BGJ398-treated cells. Representative images of tumor spheroids at 10 days are shown. The data in A, and B are expressed as percent inhibition of cell proliferation vs. control and are means ± SD of at least three independent experiments. The data in C are means ± SD of four independent determinations.

H1581R1 and H1581R2 cell clones were then analyzed for their sensitivity to UPR1371-UPR1376 in comparison with FIIN-2. As shown in Figure 6A, the irreversible reference compound slightly affected cell proliferation; among our compounds, only UPR1376 showed a marked anti-tumor activity, inhibiting cell proliferation almost completely at 1 μM.

We therefore focused our attention on UPR1376. We treated the resistant cell clones with increasing concentrations of UPR1376 and demonstrated that this compound inhibited cell proliferation in a dose-dependent manner in both H1581R1 and H1581R2 cells, with IC_50_ values of 220 and 312 nM, respectively (Figure 6B). In addition, we evaluated whether UPR1376 was effective in inhibiting cell growth also in 3D systems. As shown in Figure 6C, H1581R1 cells were capable of growing as tumor spheroids in the presence of BGJ398. FIIN-2 had no growth-inhibitory effect; in contrast, UPR1376 induced a complete block of cell growth, confirming its ability to circumvent resistance to BGJ398 in H1581-derived cells.

**Figure 7:**
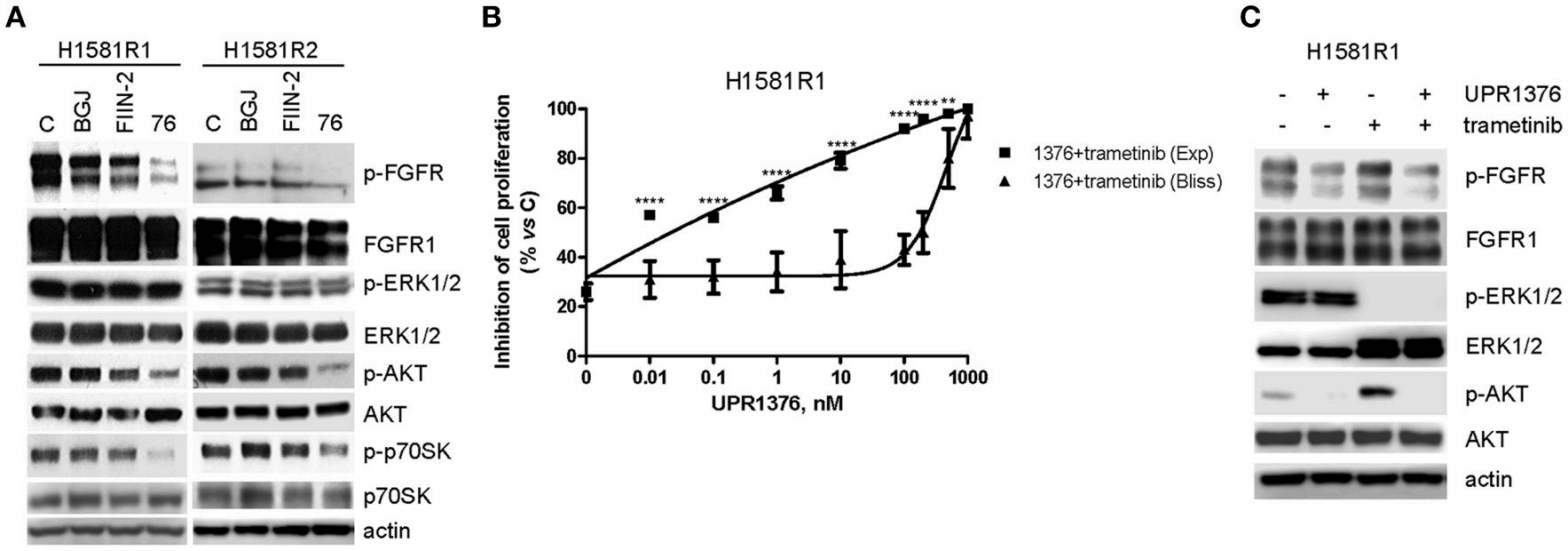
Effects of FGFR inhibitors on cell signaling and effects of UPR1376 combined with trametinib in BGJ398-resistant cell clones. **(A)** H1581R1 and H1581R2 cells were incubated in the absence or presence of 1 μM BGJ398, FIIN-2 or UPR1376. After 6 h the cells were lysed and the protein extracts were analyzed by Western blotting for the indicated proteins. **(B)** H1581R1 cells were incubated with increasing concentrations of UPR1376 (0.01–1,000 nM) in combination with 100 nM trametinib. After 72 h cell proliferation was assessed by MTT assay. The effect of the drug combination was evaluated using the Bliss interaction model. ^**^p < 0.01, ^****^p < 0.0001 vs. the corresponding points of the Bliss curve. **(C)** H1581R1 cells were incubated with 1 μM UPR1376, 100 nM trametinib or a combination of both. After 6 h the cells were lysed and the protein extracts were analyzed by Western blotting for the indicated proteins. Results are representative of three independent experiments.

Then we evaluated the effects of UPR1376 on FGFR1 signaling in comparison with BGJ398 and FIIN-2 (Figure 7A). As expected, BGJ398 did not inhibit FGFR1 nor affected downstream pathways in resistant clones. FIIN-2 marginally affected FGFR1 phosphorylation and the downstream signaling, thus justifying its low inhibitory activity on cell proliferation on H1581R clones. In contrast, UPR1376 significantly inhibited FGFR1 phosphorylation/activation in both cell clones and, to some extent, also affected downstream pathways. In particular, UPR1376 down-regulated the AKT pathway, as indicated by the significant reduction of both p-AKT and p-p70S6K levels; however, no inhibition on ERK1/2 phosphorylation was observed, suggesting that reactivation of the MAPK pathway occurred independently of FGFR1, likely due the emergence of *NRAS* amplification (38).

These findings suggest that H1581R1 and H1581R2 cell clones still rely on FGFR1 and downstream AKT signaling for their proliferation. However, it is worth noting that the resistant clones were less sensitive to UPR1376 than the parental cells (IC_50_ values of 220 nM for H1581R1 clone and 312 nM for H1581R2 clone vs. 0.8 nM for inhibitor-sensitive H1581 cells), suggesting that a contribution to cell growth in the resistant cells may also derive from MAPK signaling through FGFR1-independent mechanisms.

The persistent activation of the MAPK pathway in BGJ398-resistant cell clones provided the rationale for combining UPR1376 with trametinib, a highly specific inhibitor of MEK1/2 proteins, which are components of the MAPK signaling. H1581R1 cells were exposed to increasing concentrations of UPR1376 combined with a fixed concentration of trametinib, chosen on the basis of a dose-response curve of proliferation previously determined (not shown). According to the Bliss experimental model, such combination produced synergistic anti-proliferative effects (Figure 7B). The stronger efficacy of the combination compared to the single treatments was associated with the simultaneous down-regulation of the AKT pathway, mediated by UPR1376 through FGFR1 inhibition, and the MAPK pathway, mediated by trametinib (Figure 7C). Of note, trametinib increased AKT phosphorylation, which was completely inhibited by UPR1376 treatment. Altogether these results suggest that treatment with UPR1376 may be an effective therapeutic approach to overcome resistance to BGJ398 and that its efficacy may be further improved by combination with trametinib when the resistance is also associated with persistent activation of the MAPK pathway.

## Discussion

Aberrant activation of FGFR signaling has been demonstrated to play a key role in sustaining the growth of multiple cancers, including SQCLC, thereby offering novel opportunities for targeted therapeutic intervention. Both non-selective and selective FGFR inhibitors have shown strong anti-tumor activity in pre-clinical studies and are currently being evaluated in clinical trials for the treatment of patients with tumors carrying FGFR alterations. Although promising results are emerging from these studies, several challenges are being faced, including the selection of patients most likely to respond to FGFR inhibitors, the management of toxicity profiles, and the appearance of drug resistance (17, 22).

Recently, the development of irreversible FGFR inhibitors has received growing attention due to their increased efficiency, functional selectivity, and ability to circumvent acquired resistance (39).

In this study, starting from 1-(4-aminobenzyl)-pyrimido[4,5-*d*] pyrimidin-2-one core of FIIN-2, we synthesized a set of four inhibitors of FGFR in which the acrylamide warhead present in FIIN-2 was replaced by a 3-APA group (i.e., UPR1371), potentially able to generate an acrylamide in cancer cells (30), or by activated acetamides (i.e., UPR1372, 1373, and 1376), potentially able to alkylate the P-loop cysteine of FGFR by nucleophilic substitution. In LENTI-4 cells, a FGFR1-over-expressing SQCLC cell model generated in our lab, we found that incubation with UPR1371 failed to give persistent inhibition of FGFR1 suggesting that, in this SQCLC cell model, the conversion of the 3-APA group in acrylamide did not occur. Also the 2-(imidazol-2-ylthio)acetamide UPR1372 and the 2-(tetrazol-5-ylthio)acetamide UPR1373 failed to irreversibly inhibit FGFR1. In fact, 8 h after their removal from the treated cells, the recovery of FGFR1 activity was nearly complete, with phosphorylation levels approaching (UPR1372, UPR1373) or overcoming (UPR1371) the 90% of the control. The heteroarylthio acetamide group of UPR1372 and UPR1373 had been devised as warheads with very low reactivity; the present results show that higher reactivity is needed to get irreversible inhibition of FGFR. On the other hand, the chloroacetamide derivative UPR1376 was able to maintain FGFR1 significantly inhibited after its removal from the cells. The comparison of the auto-phosphorylation levels of FGFR at 8 h between UPR1376 (33% vs. control) and FIIN-2 (49% vs. control) indicated that the former compound was more effective than the latter in irreversibly inhibiting FGFR1. In light of these data, taking into consideration the well-known reactivity of chloroacetamides toward thiols in solution (40), and within the kinase active site (41), we speculated that the higher activity of UPR1376 may arise from a more efficient alkylation of the P-loop cysteine of FGFR1 compared to the FIIN-2 compound. Although additional experiments have to be performed to validate this hypothesis, a covalent interaction between FGFR1 and UPR1376 appears very likely. The good metabolic stability displayed by UPR1376 in cellular medium suggests that this compound might be able to engage FGFR in the malignant tissue when administered *in vivo*. On support of this, an EGFR inhibitor featured by a similar chloroacetamide warhead has been successfully used in the treatment of a mouse xenograft model of NSCLC (42).

In light of the prominent reactivity of chloroacetamide, we preliminary evaluated the toxicity of UPR1376 by testing it on SQCLC SKMES-1 cells expressing low levels of FGFR1. This compound did not affect the proliferation of SKMES-1 cells up to 1 μM (Supplementary Figure 1), similarly to what had been observed for BGJ398 and FIIN-2, indicating the selective targeting of FGFR1 and its isoforms.

UPR1376 demonstrated a significant anti-proliferative activity in H1581 NSCLC cells harboring FGFR1 amplification in both 2D and 3D systems. Most importantly, UPR1376 was shown to restore sensitivity to FGFR1 inhibition in H1581-derived cell clones generated through chronic exposure to BGJ398 and become resistant to both BGJ398 and the irreversible reference inhibitor FIIN-2.

To date, multiple mechanisms of resistance to FGFR inhibitors have been described, mostly in pre-clinical studies, which can be related to the activation of compensatory signaling or the appearance of gatekeeper mutations in the FGFR receptors themselves (14).

In H1581 cells sensitive to FGFR1 inhibition UPR1376, as well as BGJ398 and FIIN-2, inhibited either the MAPK or the AKT/mTOR pathways downstream of FGFR1. In H1581R cell clones, resistance to BGJ398 was associated with the maintenance of FGFR1 phosphorylation and with the persistent activation of both signaling cascades. The inability of BGJ398 to suppress FGFR1 activation was not due to the acquisition of the gatekeeper V561M mutation, previously shown to confer resistance to FGFR inhibitors in different cancer models (35–37). However, we cannot exclude the presence of other mutations at FGFR1 level. Recently, the increased expression of the drug efflux transporter ABCG2 has been identified as an additional mechanism of resistance to the selective FGFR inhibitor AZD4547 (43). However, it does not seem to be the case for H1581R cell clones, since the intracellular accumulation of BGJ398 in these cells is comparable to that observed in the parental cells. H1581R cell clones acquired cross-resistance also to the irreversible inhibitor FIIN-2. UPR1376, in contrast with BGJ398 and FIIN-2, significantly inhibited the phosphorylation/activation of FGFR1 and the downstream AKT/mTOR pathway, thus impairing cell proliferation. A prominent role for AKT/mTOR in FGFR signaling has been previously demonstrated in cancer in a number of studies (24, 44–46). In addition, activation of AKT in cancer cell lines carrying activating FGFR alterations has been reported as a mechanism of acquired resistance to BGJ398, which can be efficaciously reverted by treatment with an AKT inhibitor (47). In H1581R clones, not AKT but MAPK signaling was activated independently of FGFR1, being ERK1/2 phosphorylation maintained also in the presence of UPR1376-mediated inhibition of FGFR1. Interestingly we found that such up-regulation was associated with *NRAS* amplification.

The persistent activation of MAPK signaling likely contributes to BGJ398 resistance in H1581R clones, also reducing to some extent their sensitivity to UPR1376 in comparison with the parental cells. Indeed, the anti-tumor efficacy of UPR1376 was greatly improved by the combination with the specific MEK1/2 inhibitor trametinib. Recently, reactivation of MAPK pathway, mediated by *NRAS* amplification or *MET* transcriptional regulation, has been linked to the emergence of resistance to FGFR inhibitors in FGFR1-amplified lung cancer cell models (38). In these cells, co-treatment with trametinib or the MET inhibitor crizotinib restored the sensitivity to BGJ398 by inhibiting the MAPK signaling in a direct or indirect fashion, respectively.

It is worth noting that treatment with trametinib alone, while inhibiting the MAPK signaling, increased AKT phosphorylation/activation in H1581R cells, resulting in a limited efficacy; UPR1376, blocking FGFR1 signaling, completely reverted this effect leading to a synergistic impairment of cell growth. Based on these findings, it is conceivable that the simultaneous inhibition of FGFR/AKT and MAPK signaling is required to achieve a significant anti-proliferative response, overcoming the resistance to FGFR inhibitors.

Collectively our results suggest the concomitance of different mechanisms of resistance in H1581R cell clones. This is in line with the results from a recent study describing a SQCLC cell model of acquired resistance to FGFR inhibitors, in which the activation of the MET/MAPK axis co-exists with an independent change of the *AKT1* gene leading to the activation of AKT signaling (48).

These observations support the notion that the emergence of multiple genetic lesions within the same cells may represent a common mechanism of resistance requiring a combined therapy intervention to restore tumor cell responsiveness.

## Conclusions

Because of the recognized role of FGFR signaling in cancer progression, intensive efforts are being made to develop effective FGFR-targeted therapies, which are especially urgent for challenging-to-treat cancers, like SQCLC, that still have few treatment options available. In this study, among the reported compounds, chloroacetamide UPR1376 emerged as a promising irreversible inhibitor of FGFR able to block proliferation of FGFR1-amplified H1581 cells with a potency higher than BGJ398, while sparing FGFR1 low-expressing cells. Interestingly, in two distinct H1581-derived clones resistant to BGJ398, UPR1376 inhibited proliferation at nanomolar concentration, an effect that was strongly enhanced by trametinib. Collectively, our results suggest that the insertion of a chloroacetamide warhead on a suitable scaffold is a viable strategy to find a novel generation of FGFR inhibitors, which may offer new therapeutic opportunities for treating SQCLC patients with FGFR alterations and overcoming acquired resistance.

## Data Availability

This manuscript contains previously unpublished data. The name of the repository and accession number are not available.

## Author Contributions

MM, PP, and AA: conception and design; CF, MB, SL, DC, RM, and MG: cell biology and molecular biology experiments; NB, RC, and GM: chemical synthesis; FF: chemical analysis; CF and AL: writing of the manuscript; MB and RA: review of the manuscript; MM, PP, and MT: study supervision. All authors contributed to revise the manuscript and approved the final version for publication.

### Conflict of Interest Statement

The authors declare that the research was conducted in the absence of any commercial or financial relationships that could be construed as a potential conflict of interest.
