# Supplementary material for: Expanding the Arsenal of FGFR Inhibitors: A Novel Chloroacetamide Derivative as a New Irreversible Agent With Anti-proliferative Activity Against FGFR1-Amplified Lung Cancer Cell Lines
Source: Front Oncol. 2019 Mar 26;9:179. doi: 10.3389/fonc.2019.00179 (PMC6443895; doi:10.3389/fonc.2019.00179)
Supplement: Supplementary file 1 [file Data_Sheet_1.docx]

***Supplementary Material***


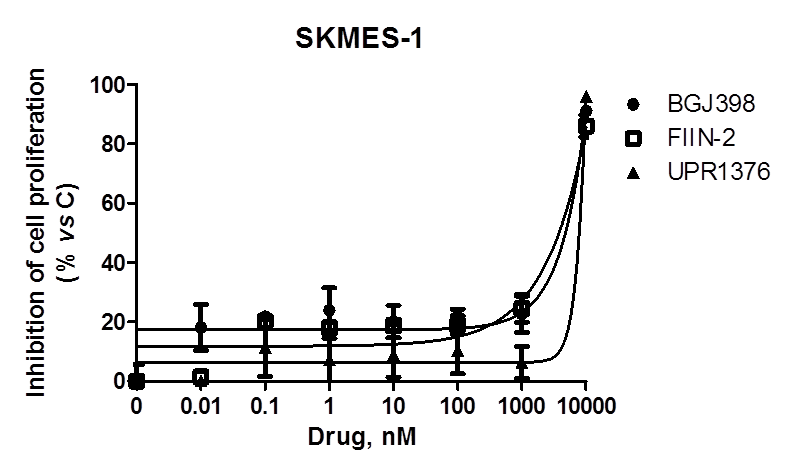
**Biology**

**Supplementary Figure 1: Effects of BGJ398 and FGFR irreversible inhibitors on cell proliferation of FGFR1-low expressing SQCLC cells.** SKEMS-1 cells were incubated with increasing concentrations of BGJ398, FIIN-2 or UPR1376 (0.01 nM-10 µM). After 72 h, cell proliferation was assessed by MTT assay. The data, expressed as percent inhibition of cell proliferation vs. control, are representative of two independent experiments.

**Chemistry**

The synthesis of **UPR1371** was performed reacting commercially available **FIIN-2** (Selleckchem) with dimethylamine in absolute ethanol. The suspension was gradually warmed until a clear solution was obtained and **UPR1371** was easily isolated by removal of the volatiles under reduced pressure.

Intermediate **1** was prepared according to the procedure reported by Tan et al.^i^, and reacted with the mixed anhydride resulting from the reaction of the trialkyl ammonium salt of chloroacetic acid with pivaloyl chloride, obtaining **2**. Compound **2** was treated with the suitable heterocyclic thiol under basic conditions, affording **UPR1372** and **UPR1373**. The final compound **UPR1376** was obtained as hydrochloride salt, treating methanolic solution of **2** with an excess of trimethylsilyl chloride.

**Supplementary Figure 2: Synthesis of FIIN-2 analogues.**

**Reagents and conditions:** *a*) Me_2_NH, abs. EtOH, 70°C, 15 min, 98% yield; *b*) chloroacetic acid, DIPEA, PivCl, THF, 1 h, yield not calculated; *c*) TMSCl, 51% yield over two steps; *d*) 2-mercapto-1-methylimidazole, NaOH, MeCN/THF, r.t., 15 h, 78% yield; *e*) 5-mercapto-1-methyltetrazole, NaOH, MeCN/THF, r.t., 15 h, 35% yield.

Reagents were obtained from commercial suppliers and used without further purification. Solvents were purified and stored according to standard procedures. Anhydrous reactions were conducted under a positive pressure of dry N_2_. Reactions were monitored by TLC, on Kieselgel 60 F 254 (DC-Alufolien, Merck). Final compounds and intermediates were purified by flash chromatography (SiO_2_ 60, 40−63 μm). Melting points were not corrected and were determined with a Gallenkamp melting point apparatus. The ^1^H NMR spectra were recorded on a Bruker 300 MHz Avance or on a Bruker 400 MHz Avance spectrometer; chemical shifts (δ scale) are reported in parts per million relative to the central peak of the solvent. ^1^H NMR spectra are reported in the following order: multiplicity, approximate coupling constant (*J* value) in hertz, and number of protons; signals are characterized as s (singlet), d (doublet), dd (doublet of doublets), t (triplet), dt (doublet of triplets), q (quartet), m (multiplet), and br s (broad signal). All tested compounds were at least 95% pure by HPLC/UV analysis.

**UPR1371** *N*-(4-((3-(3,5-dimethoxyphenyl)-7-((4-(4-methylpiperazin-1-yl)phenyl)amino)-2-oxo-3,4-dihydropyrimido[4,5-*d*]pyrimidin-1(2*H*)-yl)methyl)phenyl)-3-(dimethylamino)propanamide

FIIN-2 (9.5 mg, 0.015 mmol) is suspended in absolute ethanol (2 ml). Dimethylamine (33% in absolute ethanol, 20 μl, 0.11 mmol) is added, then the suspension is gradually warmed till the obtainment of a clear solution, which is cooled again to r.t. Ethanol is removed under reduced pressure, affording the title compound (10.0 mg, 98%) as a white powder. Mp: 117°C. ^1^H NMR (400 MHz, CDCl_3_) δ 10.80 (s, 1H), 7.93 (s, 1H), 7.50 – 7.30 (m, 6H), 7.00 (s, 1H), 6.96 – 6.82 (m, 2H), 6.47 (d, *J* = 2.2 Hz, 2H), 6.37 (t, *J* = 2.2 Hz, 1H), 5.24 (s, 2H), 4.62 (s, 2H), 3.78 (s, 7H), 3.19 (t, *J* = 5.0 Hz, 4H), 2.65 – 2.57 (m, 6H), 2.48 (dd, *J* = 6.6, 5.0 Hz, 2H), 2.36 (s, 3H), 2.34 (s, 6H).

**2** 2-Chloro-*N*-(4-((3-(3,5-dimethoxyphenyl)-7-((4-(4-methylpiperazin-1-yl)phenyl)amino)-2-oxo-3,4-dihydropyrimido[4,5-*d*]pyrimidin-1(2*H*)-yl)methyl)phenyl)acetamide

Chloroacetic acid (26.0 mg, 0.27 mmol) and DIPEA (47 μl, 0.27 mmol) are dissolved in anhydrous THF (2 ml). Pivaloyl chloride (28 μl, 0.23 mmol) is added and the mixture is stirred at r.t. for 25 min. A solution of **1** (63.0 mg, 0.11 mmol) in anhydrous THF (3 ml) is added and the mixture is stirred for 30 min before being diluted with DCM, washed with water and dried over Na_2_SO_4_. The organic layer is filtered on a short silica column (DCM/MeOH 15:1). Solution from column is either directly used in the synthesis of **UPR1376** or concentrated under reduced pressure at room temperature. The obtained solid residue is directly used in the synthesis of **UPR1372** and **UPR1373**.

**UPR1376** 2-chloro-*N*-(4-((3-(3,5-dimethoxyphenyl)-7-((4-(4-methylpiperazin-1-yl)phenyl)amino)-2-oxo-3,4-dihydropyrimido[4,5-*d*]pyrimidin-1(2*H*)-yl)methyl)phenyl)acetamide hydrochloride

TMSCl (17 μl, 0.13 mmol) is added to the solution obtained from the synthesis of **2**. The mixture is concentrated under reduced pressure at low temperature, affording the title compound (37 mg, 51%, yield calculated over two steps) as a bright yellow solid. Mp: 85°C (dec.). ^1^H NMR (400 MHz, CD_3_OD) δ 7.95 (s, 1H), 7.46 (d, *J* = 8.2 Hz, 2H), 7.33 (d, *J* = 8.5 Hz, 2H), 7.28 (d, *J* = 8.2 Hz, 2H), 6.88 (d, *J* = 8.5 Hz, 2H), 6.48 (d, *J* = 1.9 Hz, 2H), 6.40 (t, *J* = 2.3 Hz, 1H), 5.21 (s, 2H), 4.69 (s, 2H), 4.10 (s, 2H), 3.77 (s, 6H), 3.21-3.15 (m, 4H), 2.77-2.70 (m, 4H), 2.43 (s, 3H).

**UPR1372** *N*-(4-((3-(3,5-dimethoxyphenyl)-7-((4-(4-methylpiperazin-1-yl)phenyl)amino)-2-oxo-3,4-dihydropyrimido[4,5-d]pyrimidin-1(2H)-yl)methyl)phenyl)-2-((1-methyl-1H-imidazol-2-yl)thio)acetamide

Compound **2** (21.5 mg, 0.03 mmol) is dissolved in MeCN/THF 1:1 (4 ml). A solution of 2-mercapto-1-methylimidazole (7.5 mg, 0.07 mmol) and NaOH (3.0 mg, 0.07 mmol) in THF (0.4 ml) is added and the mixture is stirred at r.t. for 15 h. The mixture is diluted with DCM and washed with saturated aqueous NaHCO_3_ and brine, dried over Na_2_SO_4_ and concentrated under reduced pressure. The residue is purified by silica gel column chromatography (DCM/MeOH 15:1) to afford the title compound (19 mg, 78%) as a white solid. Mp: 112°C. ^1^H NMR (400 MHz, CDCl_3_) δ 11.02 (s, 1H), 7.93 (s, 1H), 7.46 (d, *J* = 8.1 Hz, 2H), 7.41 – 7.30 (m, 4H), 7.07 (s, 1H), 6.97 (s, 1H), 6.91 (d, *J* = 2.7 Hz, 2H), 6.89 (s, 1H), 6.47 (d, *J* = 2.1 Hz, 2H), 6.38 (t, *J* = 2.2 Hz, 1H), 5.23 (s, 2H), 4.62 (s, 2H), 3.78 (d, *J* = 1.3 Hz, 6H), 3.75 (s, 2H), 3.57 (d, *J* = 1.4 Hz, 3H), 3.21 (t, *J* = 5.1 Hz, 4H), 2.64 (t, *J* = 5.2 Hz, 4H), 2.39 (d, *J* = 1.7 Hz, 3H).

**UPR1373** *N*-(4-((3-(3,5-dimethoxyphenyl)-7-((4-(4-methylpiperazin-1-yl)phenyl)amino)-2-oxo-3,4-dihydropyrimido[4,5-*d*]pyrimidin-1(2*H*)-yl)methyl)phenyl)-2-((1-methyl-1*H*-tetrazol-5-yl)thio)acetamide

Compound **2** (35.0 mg, 0.05 mmol) is dissolved in MeCN/THF 1:1 (4 ml). A solution of 5-mercapto-1-methyltetrazole (13.0 mg, 0.11 mmol) and NaOH (4.5 mg, 0.11 mmol) in THF (0.45 ml) is added and the mixture is stirred at r.t. for 15 h. The mixture is diluted with DCM and washed with saturated aqueous NaHCO_3_ and brine, dried over Na_2_SO_4_ and concentrated under reduced pressure. The residue is purified by silica gel column chromatography (DCM/MeOH 15:1) to afford the title compound (13 mg, 35%) as a white solid. Mp: 145°C (dec.). ^1^H NMR (400 MHz, CDCl_3_) δ 9.33 (s, 1H), 7.94 (s, 1H), 7.41 (d, *J* = 8.2 Hz, 2H), 7.38 – 7.29 (m, 4H), 6.90 (d, *J* = 9.0 Hz, 2H), 6.47 (d, *J* = 2.2 Hz, 2H), 6.37 (t, *J* = 2.3 Hz, 1H), 5.21 (s, 2H), 4.76 – 4.51 (m, 2H), 4.01 (s, 2H), 3.94 (s, 3H), 3.77 (s, 6H), 3.28 – 3.11 (m, 4H), 2.64 (t, *J* = 5.0 Hz, 4H), 2.39 (s, 3H).

**LC-MS analytical method for BGJ398, FIIN-2 and UPR1376 dosage**

Calibration curves of BGJ398, FIIN-2 and UPR1376 were prepared by spiking into control matrices the stock solutions of test compounds, freshly prepared in DMSO, to yield final concentrations in the 1000-1 nM concentration range. Chromatographic separation was achieved on a Phenomenex Synergi Fusion C18 column (100 × 2.1 mm i.d., 3 μm particle size; Phenomenex, USA). Mobile phases consisted of water as eluent A and acetonitrile as eluent B, both added with 0.1% v/v formic acid and at a flow rate of 350 μL min^-1^. A linear gradient elution was set up: T(0 min): 95% A: 5% B; T(1 min): 95% A: 5% B; T(6 min) 5% A: 95% B; T(8 min) 5% A: 95% B returning to initial conditions after 1 min, followed by 3 min re-equilibration time. A Thermo TSQ Quantum Access Max triple quadrupole mass spectrometer (Thermo, San Jose, CA, USA) equipped with a heated electrospray ionization (H-ESI) source was employed for compound detection and analysis, acquiring in positive ion (ESI+) and multiple reaction monitoring (MRM) mode. H-ESI interface parameters: probe middle (D) position; capillary temperature: 270°C; spray voltage: 3.5 kV. Nitrogen was employed as nebulizing gas at the following pressure: sheath gas: 35 psi; auxiliary gas 15 arbitrary units (a.u.). Argon was used as the collision gas at a pressure of approximately 1.5 mtorr (1 torr = 133.3 Pa). For quantitative analysis, the following parent→product ion transitions were selected: **FIIN-2**: m/z 635.2 [M+H]^+^ → m/z 297.1 + m/z 309.1 + m/z 476.3 (Tube Lens (TL) 117 V; Collision Energies (CE) 43, 29, 31 eV, respectively); **BGJ398**: m/z 560.1 [M+H]+ → m/z 311.1 + m/z 313.1 + m/z 339.1 (TL 83 V; CE 30, 22, 24 eV); **UPR1376**: m/z 657.2 [M+H]+ → m/z 297.0 + m/z 309.1 + m/z 476.3 (TL 127 V; CE 45, 29, 33 eV), with a scan time of 0.15 s per transition. Data acquisition, peak integration and calibration curves was analyzed by Thermo Xcalibur software version 1.3 (Thermo, USA).

^i^Tan L, Wang J, Tanizaki J, Huang Z, Aref AR, Rusan M, et al. Development of covalent inhibitors that can overcome resistance to first-generation FGFR kinase inhibitors. *Proc Natl Acad Sci U S A* (2014) 111(45):E4869-77. Epub 2014/10/29. doi: 10.1073/pnas.1403438111

**Evaluation of chemical stability of FGFR inhibitors in cellular medium**

The chemical stability of BGJ398, FIIN-2, and UPR1376 in cellular medium was assessed by spiking the stock solution of the compound of interest in dimethylsulfoxide (DMSO) into the medium, to a final concentration of 100 µM (final DMSO percentage: 1 %). Right after the addition of the stock in the medium (t = 0 min) and after 8 h of co-incubation (t = 8 h), an aliquot of the sample was withdrawn and diluted into a 100 nM solution of the IS in acetonitrile. The resulting sample was injected into the HPLC-MS system for analysis. The ratio between the compound of interest and the IS at the starting point was considered as 100 % and the chemical stability of each FGFR inhibitor was measured as the residual percentage of the ratio at t = 8 h.

In details, data acquisition was performed by using a Thermo Accela U-HPLC system equipped with an Accela Open AS autosampler interfaced to a TSQ Quantum Access Max triple quadrupole mass spectrometer (Thermo, Milan, Italy) with a heated electrospray ionization (H-ESI) ion source. Mass spectrometric analyses were carried in positive ion mode and H-ESI interface parameters were set as follows: probe in middle (D) position; capillary temperature at 270 °C; spray voltage at 3.5 kV. Nitrogen was used as nebulizing gas at the following pressure: sheath gas 35 psi; auxiliary gas: 15 arbitrary units (a.u.). Argon was used as the collision gas at a pressure of approximately 1.5 mtorr (1 torr = 133.3 Pa).

The following parent ion 🡪 product ion transitions were optimized and monitored: m/z = 560.1 [M+H]+ 🡪 m/z = 311.1, 313.2, 339.1 (tube lens (TL): 83 V; collision energies (CE): 30, 22, 24 eV, respectively) for BGJ398; m/z = 635.2 [M+H]+ 🡪 m/z = 297.1, 309.1, 476.3 (tube lens (TL): 117 V; collision energies (CE): 43, 29, 31 eV, respectively) for FIIN-2; m/z = 657.2 [M+H]+ 🡪 m/z = 297.0, 309.1, 476.3 (tube lens (TL): 127 V; collision energies (CE): 45, 29, 33 eV, respectively) for UPR1376. FIIN-2 was employed as IS for both BGJ398 and UPR1376 that was used, in turn, as IS for FIIN-2.

A Phenomenex Synergi Fusion C18 column (100×2.1 mm; 4µm particle size) was employed for compound separation following a gradient elution. Flow rate was set at 0.350 mL·min^-1^. The following solvent were used: solvent A: water and solvent B: acetonitrile both added with 0.1% v/v formic acid. HPLC gradient was as follows: t(0 min) A: 90%, B: 10%; t(5 min): A: 5%, B: 95%; t(7 min): A: 5%, B: 95%; t(7.5 min): A: 90%, B: 10% followed by 2.5 min of column reconditioning. Retention times were: 3.23 min for BGJ98; 3.04 min for FIIN-2 and 3.09 min for UPR1376

Data acquisition and processing were performed by Thermo Xcalibur software v. 2.1 (Thermo, Milan, Italy).
